# Does mistreatment during institutional childbirth increase the likelihood of experiencing postpartum depressive symptoms? A prospective cohort study in Nepal

**DOI:** 10.1080/16549716.2024.2381312

**Published:** 2024-07-31

**Authors:** Rejina Gurung, Miia Bask

**Affiliations:** a Department of Women’s and Children’s Health, Uppsala University, Uppsala, Sweden; b Research Division, Golden Community, Lalitpur, Nepal; c Department of Sociology, Uppsala University, Uppsala, Sweden

**Keywords:** Postpartum depressive symptoms, institutional birth, mistreatment during childbirth, respectful maternity care, Nepal

## Abstract

**Background:**

Postpartum depression is associated with low socioeconomic status, adverse birthing processes, and life stress. Increasing evidence of mistreatment during childbirth, negative birth experiences, and poor quality of maternal care is of global concern.

**Objective:**

To assess the risk of experiencing depressive symptoms among postpartum women exposed to mistreatment during institutional birthing in Nepal.

**Method:**

We conducted a prospective cohort study from 29 March to 19 August 2022. Of 1629 women who gave birth in a hospital in Nepal, 1222 were assessed for mistreatment during childbirth and depressive symptoms using the Edinburgh Postnatal Depression Scale. We used binomial generalized linear mixed model to examine the risk ratio of postpartum depressive symptoms in women exposed to mistreatment during childbirth.

**Results:**

The prevalence of postpartum depressive symptoms was 4.4%. Women exposed to mistreatment during childbirth were almost fifty percent more likely to have postpartum depressive symptoms (cRR 1.47; 95% CI 1.14, 1.89; *p* = 0.003) compared with the unexposed group. Furthermore, adolescent mothers exposed to mistreatment during childbirth had a seventy percent increased risk of depressive symptoms (aRR 1.72; 95% CI 1.23, 2.41; *p = 0.002*). Similarly, women who gave birth to female infants were thirty percent more likely to experience postpartum depressive symptoms (aRR 1.32; 95% CI 1.01–1.74; *p = 0.039*).

**Conclusion:**

We observed an association between postpartum depressive symptoms and mistreatment during institutional births in Nepal. The implementation of appropriate respectful maternity care during childbirth and also routine screening for depressive symptoms is critical to improving perinatal mental health and well-being.

## PAPER CONTEXT

- **Main findings:** Despite widespread reports of mistreatment during childbirth in health facilities, little is known about possible association with poor perinatal mental health outcomes.
- **Added knowledge:** This prospective cohort study in Nepal found an association between postpartum depressive symptoms and mistreatment during institutional childbirth.
- **Global health impact for policy and action:** Postpartum depression is a global health issue, and there is an urgent need for interventions to promote respectful maternity care.

## Background

The global/ prevalence of postpartum depression is 17.2%. In low- and middle-income countries, almost one in five women experience postpartum depression [1,2]. Common risk factors are socioeconomic disparity, low educational attainment, being nulliparous, alcohol use during pregnancy, history of psychiatric illness, stressful life events, poor intimate relationships, and adverse reproductive outcomes [2–5]. Persistent mood disorders in the postpartum period have serious consequences, including severe mental illness and suicide attempts [6–8]. In addition, the progression of postpartum depression jeopardizes early mother–child interaction and results in delayed infant growth and development [3,5,9].

Nepal’s National Mental Health Survey (NMHS), conducted between January 2019 and January 2020, reported a prevalence of 1.4% for current mood disorders in the adult population. Major depressive disorder (4%) and suicide attempts were found to be higher in reproductive aged Nepali women than in men of comparable age [10]. Studies in Nepal have reported a prevalence of postpartum depression ranging from 4.9% to 37% [3–5,9,10]. A situational analysis in Nepal suggests a low prevalence in community surveys, while facility-based studies have reported a high prevalence of postpartum depression [2]. Studies in Nepal have identified social and economic inequality, pregnancy-related health problems, multiple births, sex of the baby, maternal age, and caesarean section as contributing factors to postpartum depression [5,11–14]. A multi- country study found that the lack of routine mental health screening during pregnancy and postnatal period delays early identification and treatment of perinatal mental health problems [15].

Mistreatment in facility-based births is highly prevalent worldwide [6,16–21]. Mistreatment during childbirth has been identified as non-dignified care [7], verbal or physical abuse [8,19,21–24], unnecessary medical interventions [21], poor communication between the woman and her provider [24], lack of companionship at birth [21], negligence by providers [21,22,24] and breach of confidentiality [25]. Subjective perceptions of labor pain, dissatisfaction with social support, and disrespectful behavior by health workers can lead to negative birth experiences among pregnant women [26,27]. The literature suggests that negative birth experiences can lead to lifelong shame and self-blame in women [28–30], and some studies have linked this to depressive disorders [31–36]. Furthermore, the experience of poor quality of care, compounded with mistreatment and lack of emotional support during childbirth, could lead to adverse maternal and newborn health outcomes [20,37–40]. Mistreatment during childbirth is associated with birth complications and low future utilization of maternal health service [7,41,42].

The most recent Nepal Demographic Health Survey (2022) reported that 79% of births in Nepal took place in health facilities. This figure represents a tenfold increase in births since 1996, with increasing trends in skilled attendance at birth and decreasing neonatal mortality [43]. Over the past decade, maternal mortality has reduced significantly from 349 to 174 [43,44]. However, there are increasing reports of mistreatment during childbirth in Nepalese health facilities, including care provision without proper consent, unnecessary medical interventions, lack of birth companionship, unclear and incomplete information from providers, lack of privacy, delays in service, and physical and verbal abuse [45–50]. Other associations of mistreatment have been found with ethnicity, number of previous births, maternal age, and length of hospital stay [45,46,50].

The World Health Organization (WHO) Quality of Care Framework for Maternal and Newborn Health clearly states that a positive birth experience is as important as the provision of quality care in achieving person-centered positive health outcomes [51]. As the rate of institutional births in Nepal increases [43], it can be assumed that the likelihood of women experiencing mistreatment during childbirth will also rise. Furthermore, national mental health policies in Nepal focus on reducing major depressive disorders in adolescents and the female population in general [52], while depressive symptoms in postpartum women receive less attention, despite their high prevalence of suicidal ideation [10,53]. Due to limited evidence of the adverse maternal health outcomes associated with mistreatment during institutional childbirth in Nepal, it is timely to investigate these issues. This study assesses the risk of experiencing postpartum depressive symptoms among postpartum women exposed to mistreatment during institutional birthing in Nepal.

## Methods

### Study design

A prospective cohort study was conducted from 29 March to 19 August 2022. Of the 1629 enrolled participants, 1222 women who gave birth in a public hospital between 15 January and 14 May 2022 were followed up by telephone. Study participants were interviewed once at different points in time between 6 and 24 weeks postpartum.

## Study setting

The study site is a 600-bed public hospital located in Bharatpur district, in the central-southern part of Nepal [54]. This Bharatpur hospital has over 13,000 births per year, of which approximately 40% are by cesarean section [47,54]. The hospital also offers a vacuum-assisted delivery service. The hospital provides free maternal and newborn health services to women from diverse ethnic backgrounds [54–57]. Routine and referral services are provided by a team of Skilled Birth Attendant (SBA) nurses, obstetricians, and pediatricians. Licentiate nurses with an academic background at diploma and bachelor level undergo a two-month training course to become SBAs [56]. This training includes managing obstetric complications such as vacuum delivery, postpartum hemorrhage, eclampsia, and neonatal asphyxia.

We purposively selected Bharatpur hospital for the study because it is a tertiary care center with a high volume of annual deliveries, serving a diverse population of women from various socio-demographic backgrounds.

## Inclusion criteria

Participants who were at least 15 years of age and/or had a vaginal birth, including a vacuum delivery, or had undergone an emergency cesarean section were eligible and included in the study after obtaining informed consent. Women who had undergone elective caesarean sections, had a stillbirth, were first-degree relatives of hospital staff, or had been diagnosed with mental illness during or before pregnancy were excluded from the study.

## Data collection and data source

Licentiate nurses from the local area were recruited and trained to collect quality data. The independent data collection team had no previous or current clinical affiliation with the hospital. Information on socio-demographics, birth history, current obstetric characteristics, and diagnosed mental illness was collected from patient records and birth registers. Participants were contacted at the end of their sixth postpartum week. First contact calls were made to verify the contact details, conduct eligibility screening, and obtain informed verbal informed consent from those who agreed to participate in the study. During the first contact call, appointments for assessments were made, and participants were asked to choose a private space for the interview. As study participants had given birth at different times, data collection by telephone interviews began chronologically after the completion of the 6-week postpartum period. Data collection took place at different weeks in the postnatal period between 6 and 24 weeks, based on contact calls with participants and assessment appointments. A few participants rescheduled and attended the interview at 25 weeks postpartum. Repeat calls were made every three days to women who did not receive a call or in cases where contact numbers were unreachable during the first contact call. These calls were made at different times of the day to increase the likelihood of contacting participants. Loss to follow-up was confirmed if contact could not be established after four attempted calls over a two-week period.

A digital tablet-based form was used for data collection, consisting of: a screening form to include eligible participants; an extraction form to collect obstetric and co-morbid related information from hospital records; a modified structured mistreatment questionnaire to collect reports of mistreatment; and assessments based on the Edinburgh Postnatal Depression Scale (EPDS). The mistreatment and EPDS assessments were administered simultaneously during telephone interviews.

## Data collection tool

The extraction form was adapted from quality improvement research conducted in similar settings [58] to collect participants’ sociodemographic and obstetric history. This information was obtained from hospital registers. The mistreatment assessment questionnaire was developed through an iterative process of literature review and expert opinion. The questionnaire included items such as physical abuse, verbal abuse, stigma and discrimination, vaginal examination, presence of a birth companion, negligence, autonomy, and medical intervention. The questionnaire was translated into Nepali and cognitive testing was carried out on some postnatal women who were not part of the study. The cognitive testing provided valuable insights into language comprehension and the vocabulary of the tool.

The EPDS was used to assess postpartum depressive symptoms. The EPDS is a freely available, simple 10-item scale that can be used by non-psychiatric trained professionals to assess depressive symptoms in the postpartum period. The EPDS tool has been validated and is commonly used for research in Nepal [59].

## Sample size

In Nepal, the prevalence of major depressive disorder (MDD) in the female population is 4% [60]. We used the validation of the EPDS with the DSM-IV (Diagnostic and Statistical Manual for Mental Disorders-IV) as a benchmark to determine the sample size, which showed that 12% of the population had depressive symptoms [61]. To improve both sensitivity and positive predictive value, we adjusted our assumption to 9.10%, thereby increasing the sample to achieve a better positive predictive value of 62%. We assumed that the prevalence of postpartum depressive symptoms among women exposed to mistreatment during childbirth would be 9.10% at 5% level of significance, with 80% power. We calculated 1192 as the desired sample size. With an additional attrition rate of 30%, the total sample size was estimated to be 1550 [62]. In this study, 1222 of the 1629 study participants were successfully enrolled after accounting for unreachable contacts and non-response.

### Outcome measurement

The outcome was the presence of depressive symptoms between 6 and 24 postpartum weeks. A participant with an EPDS score 9 or more was defined as suffering from depressive symptoms [63,64].

### Primary exposure

The primary exposure, mistreatment during childbirth, was based on the typology recommended by Bohren and colleagues [20]. Five types were included within the mistreatment typology (Table 1). The first type, ‘Disrespect and abuse’, was measured by two indicators, the second type, ‘Medical care and interventions’, was measured by five indicators, the third type, ‘Childbirth’, was measured by four indicators, and the fifth type, ‘Neglect’, was measured by seven indicators. Any of the following birth experiences reported by more than 7% of participants were considered mistreatment during childbirth in this study (Supplementary table S1).

**Table 1.** Primary exposure variables.

Disrespect and Abuse
– Experience of any physical abuse and verbal abuse
– Experience of stigma or discrimination
Medical care and interventions
– Painful experience of vaginal examination
– Breach of confidentiality by health care providers
– Non-consented medical interventions
– Painful episiotomy
– Painful perineal or suture repair
Mobilization, fluid and companion
– No access to water or other fluids
– Not allowed to walk or move during labor
– Not allowed to eat during labor
– Not allowed to have labor companion during labor and childbirth
Childbirth
– Health care providers not speaking preferred language
– Lack of privacy
– Not allowed to have preferred birthing position
– Absence of health care provider during admission and when baby came out
Neglect
– Felt ignored by health care providers
– Felt not emotionally supported by health care providers
– Health care provider did not listen to client’s concerns
– Health care provider did not respond to client’s questions
– Long periods of waiting time before being attended by health workers
– Shared beds with another women
– Detained in the hospital due to inability to pay hospital bills

### Co-variates

The covariates were sociodemographic factors and current obstetric characteristics. Block I includes socio-demographic variables such as maternal ethnicity and maternal employment status; block II represents current obstetric variables such as pregnancy-related complications, mode of birth, newborn complications; and block III includes both socio-demographic and obstetric variables (Table 2).

**Table 2.** Co-variables.

Maternal age	≤19 years, 20–29 years and ≥30 years	Block I	Block III
Maternal ethnicity	*Relatively advantageous*- Brahmin/Chhetri; Relatively *disadvantageous-* Janajati, Dalit, Madhesi, Muslim		
Maternal educational level	≤9 years of formal education ≥ 10 years of formal education		
Maternal employment status	Yes/No	Block I	Block III
Maternal parity	Number of previous births (0 and ≥ 1)		
Pregnancy-relatedcomplications	Yes/No/Don’t know		
Mode of birth	Vaginal and emergency caesarean	Block II	Block III
Sex of infant	Male/Female		
Newborn complication	Yes/No/Don’t know		
Breastfeeding within 1 hour after birth	Yes/No		

## Statistical analyses

Data analysis was performed using IBM SPSS Statistics version 26 and STATA 2.1.1. Initially, we calculated the prevalence of individual indicators within each typology into dichotomous variables (yes or no, agree or disagree) [19]. Then, indicators were then combined into a single indicator (yes/no) for mistreatment during childbirth with exposure to one or more types of mistreatments and with no exposure to mistreatment [32]. An EPDS score of 9 or more was categorized as depressive symptoms. The EPDS cut-off value of either 9 or more was based on validation of the EPDS tool for detection of high true positives and positive predictive values [59,63]. Descriptive statistics were used to report socio-demographic and obstetric characteristics as means, standard deviations (SD), numbers (n), and percentages (%). The chi-square test and independent sample t tests with 95% confidence intervals were used to examine descriptive data. Associations between postpartum depressive symptoms and mistreatment during childbirth, sociodemographic factors, and current obstetric characteristics were measured using binary generalized linear models and presented as crude risk ratios with 95% confidence intervals (CI).

**Figure 1.**
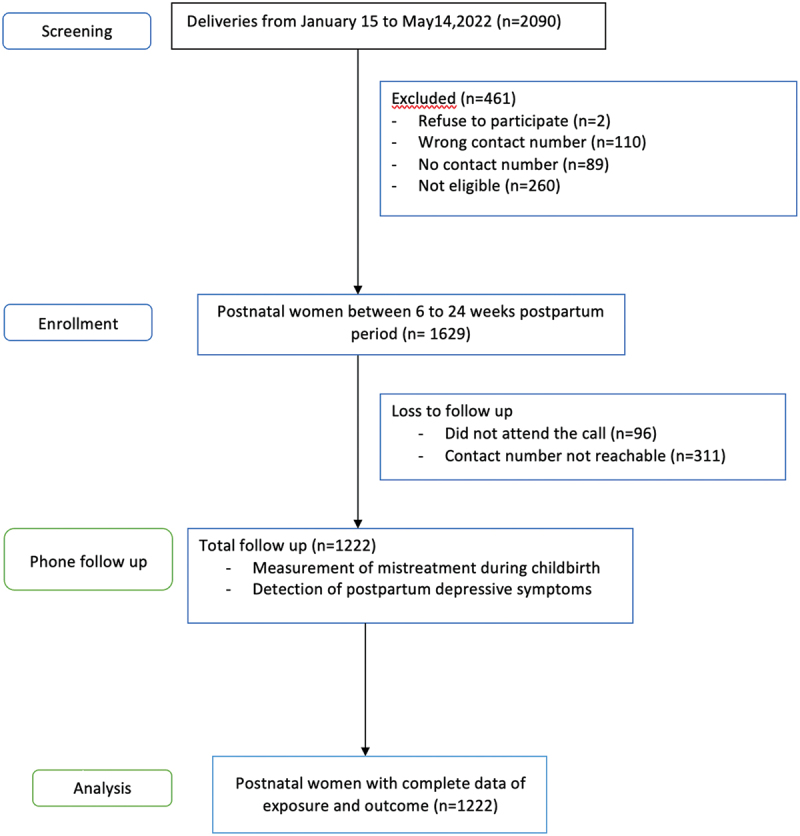
Study participants’ flowchart.

A total of 2090 women were screened for eligibility, of whom 361 were excluded from the study due to unavailability of contact details and failure to meet the eligibility criteria. Of the 1629 eligible participants, 1222 were successfully followed up for the assessments, and the same participants were included in the final analysis (Figure 1). Socio-demographic factors and current obstetric characteristics were considered confounding variables influencing both exposure and outcome [3–5,11,12,14]. Further modelling was conducted to examine the respective impacts of confounding variables (age, employment, mode of birth, sex of infant and newborn complications) on the association between postpartum depressive symptoms and mistreatment during childbirth in three blocks. In block I, the association was adjusted with socio-demographic factors. In block II, obstetric characteristics were included, and in block III, combined socio-demographic factors and obstetric characteristics were considered. Pregnancy-related complications were not included in the adjustment for current obstetric characteristics to avoid possible collinearity with the mode of birth. Associations were demonstrated as adjusted risk ratios with 95% CI, with non-depressive symptoms as reference.

Approximately, 60% of the individuals were interviewed within 15 weeks postpartum, and a sensitivity check showed that responses before and after 15 weeks did not significantly differ from each other. Missing values were less than 5% for all variables, and there were no imputations. The loss to follow-up was 33%. However, the distribution of socio-demographic, birth history, and current obstetric characteristics was similar to that of the study participants (Supplementary table S3).

## Ethical approval

Ethical approval for the study was received from the Institutional Review Board of the hospital on 7 February 2022 (Ref no. 078/79–019). Although not specifically recommended by the Ethics Board, the research team followed the protocol of contacting individual participants with high EPDS scores. Those women were then given contact details for psychiatric in-hospital care as well as counselling support from a non-governmental organization working in the area.

## Results

Of the total enrolled participants, 71.8% (874/1222) were aged 20–29 years of age, 12.6% (154/1222) were of Dalit ethnicity, 14.56% (178/1222) had pregnancy-related complications and 90.7% (1104/1222) were not in paid employment.

The prevalence of postpartum depressive symptoms was 4.4%. Regarding the socio-demographic and obstetric distribution of participants with postpartum depressive symptoms, women who gave birth to female infants experienced more postpartum depressive symptoms than those who gave birth to male babies (5.8% vs 3.3%, *p-value = 0.034*), and adolescent mothers had a stronger association with postpartum depressive symptoms (10.1% vs 3.6%vs 3.9%, *p-value = 0.003*) compared to those aged 20–29 years (Table 3).

**Table 3.** Socio-demographic and obstetric characteristics of total sample and by postnatal depressive symptoms.

*N* = 1222	Depressive symptoms		*p*-value
	Yes n (%) 54 (4.4%)	No n (%) 1168 (95.6%)	
**Maternal age (years)**			*0.425*
Mean ± SD	23.8 ± 4.8	25.0 ± 4.6	
Maternal age group			***0.003***
≤19 (139)	14(10.1%)	125(89.9%)	
20–29 (877)	32(3.6%)	845(96.4%)	
≥30 (206)	8(3.9%)	198(96.1%)	
**Ethnicity**			*0.722*
Brahmin/Chhetri *relatively advantageous (429)*	15(3.5%)	414(96.5%)	
Janajati *relatively disadvantageous (558)*	29(5.2%)	529(94.8%)	
Dalit *relatively disadvantageous (154)*	7(4.5%)	147(95.5%)	
Madhesi *relatively disadvantageous (65)*	2(3.1%)	63(96.9%)	
Muslim *relatively disadvantageous (16)*	1 (6.3%)	15(93.8%)	
**Maternal education (years)**			
≤9 (628)	32(5.1%)	596(94.9%)	*0.237*
≥10 (594)	22(3.7%)	572(96.3%)	
**Maternal employment**			
No (1108)	48(4.3%)	1060(95.7%)	*0.645*
Yes (114)	6(5.3%)	108(94.7%)	
**Previous birth**			
0 (658)	24(3.6%)	634(96.4%)	*0.165*
≥1 (564)	30(5.3%)	534(94.7%)	
**Pregnancy-induced complication**			
No (1041)	44(4.2%)	997(95.8%)	*0.414*
Yes (179)	10(5.6%)	169(94.4%)	
Don’t know (2)			
**Mode of birth**			
Vaginal (974)	46(4.7%)	928(95.3%)	*0.306*
Emergency cesarean (248)	8(3.2%)	240(96.8%)	
**Sex of baby**			
Male (691)	23(3.3%)	668(96.7%)	***0.034***
Female (531)	31(5.8%)	500(94.2%)	
**Newborn complications**			
No (1140)	47(4.1%)	1093(95.9%)	*0.149*
Yes (79)	6(7.6%)	73(92.4%)	
Don’t know (3)			
**Initial Breastfeeding**			
Within 1-hour of birth (652)	25(3.8%)	627(96.2%)	
Within 24-hour of birth (286)	15(5.2%)	271(94.8%)	
After 24-hour of birth (275)	11(4.0%)	264(96.0%)	

More than 7% of study participants reported instances of verbal abuse, limited birth companionship, feeling ignored by health workers, feeling that their presence was a nuisance to health workers and that their concerns or questions were not addressed by health workers (Supplementary Table S1). One-third of participants (*n* = 360, 29.5%) reported being mistreated in hospital during childbirth (Supplementary Table S2). Participants who experienced being ignored by health care providers were more likely to have depressive symptoms than those who did not feel ignored (10.5% vs 3.9%, *p-value = 0.003*). Similarly, participants who perceived that their presence during childbirth was a ‘nuisance’ to health care providers had an increased risk of postpartum depressive symptoms compared to others (11.4% vs 4%, *p-value = 0.002*) (Table 4).

**Table 4.** Distribution of women’s experiencing mistreatment during childbirth on postpartum depressive symptoms.

*N* = 1222	Depressive Symptoms		*p*-value
	Yes n(%) 54 (4.4%)	No n(%) 1168 (9 5.6%)	
**Experience of one or more mistreatment**			
No (857)	28(3.3%)	829(96.7%)	***0.002***
Yes (360)	26(7.2%)	334(92.8%)	
Don’t know (5)	0	5(100%)	
**Verbal abuse**			
No (1124)	48(4.3%)	1076(95.7%)	*0.392*
Yes (98)	6(6.1%)	92(93.9%)	
**Allowed to move during labour**			
Yes (1136)	47(4.1%)	1089(95.9%)	*0.082*
No (86)	7(8.1%)	79(91.9%)	
**Birth companion during labour**			
Yes (1137)	47(4.1%)	1090(95.9%)	*0.076*
No (85)	7(8.2%)	78(91.8%)	
**Received emotional support from hospital staff**			
Agree (1082)	46(4.3%)	1036(95.7%)	*0.818*
Disagree (128)	6(4.7%)	122(95.3%)	
Don’t know (12)	2(16.7%)	10(83.3%)	
**Hospital staffs listened to women’s concern**			
Agree (1101)	45(4.1%)	1056(95.9%)	*0.077*
Disagree (106)	9(8.5%)	97(91.5%)	
Don’t know (15)	0	15(100%)	
**Hospital staffs responding to women’s concern**			
Agree (1115)	45(4.0%)	1070(96.0%)	*0.111*
Disagree (93)	7(7.5%)	86(92.5%)	
Don’t know (14)	2(14.3%)	12(85.7%)	
**Long waiting time to receive care from staff**			
Disagree (1137)	49(4.3%)	1088(95.7%)	*0.668*
Agree (81)	5(6.2%)	76(93.8%)	
Don’t know (4)	0	4(100%)	
**Felt ignored by hospital staffs**			
Disagree (1124)	44(3.9%)	1080(96.1%)	***0.003***
Agree (95)	10(10.5%)	85(89.5%)	
Don’t know (3)	0	3(100%)	
**Felt nuisance to hospital staffs**			
Disagree (1137)	45(4.0%)	1092(96.0%)	***0.002***
Agree (79)	9(11.4%)	70(88.6%)	
Don’t know (6)	0	6(100%)	

On further risk analysis, women had a 1.47-fold increased risk of postpartum depressive symptoms (cRR 1.47; 95% CI 1.14, 1.89; *p = 0.003*) if they were exposed to mistreatment during childbirth compared with unexposed women. After adjustment for socio-demographic factors (maternal age and maternal employment) in block I, the adjusted risk of depressive symptoms remained 1.48 times higher for exposure to mistreatment during childbirth than for no exposure (aRR^a^ 1.48; 95% CI; 1.14, 1.92; *p = 0.003*). Similarly, when mistreatment during childbirth was adjusted for current obstetric characteristics in block II (mode of birth, sex of infant and neonatal complication), the adjusted risk of postpartum depressive symptoms remained 1.50 times higher in participants exposed to mistreatment than in the unexposed group (aRR^b^ 1.50, 95% CI, 1.15–1.95; *p = 0.002*). After adjustment for both sociodemographic factors and current obstetric characteristics in block III, the association remained significant (aRR^c^ 1.50,95% of CI, 1.15,1.96; *p = 0.003*). In addition, women younger than 19 years were 1.72 times more likely to have postpartum depressive symptoms if they had been exposed to mistreatment during childbirth (aRR^c^ 1.72, 95% CI, 1.23,2.41, *p = 0.002*) compared to women aged 20–29 years. Similarly, when mistreatment during childbirth was adjusted for having a female infant, women were 1.32 times more likely to experience postpartum depressive symptoms than those who gave birth to male infants (aRR^c^ 1.32, 95% CI, 1.01,1.74, *p = 0.039*) (Table 5).

**Table 5.** Socio-demographic and obstetric characteristics associated with postnatal depressive symptoms.

	Postnatal Depressive symptoms			
	cRR (95% CI) *P-value*	aRR^a^ (Block I) (95% CI) *P-value*	aRR^b^ (Block II) (95% CI) *P-value*	aRR^c^ (Block III) (95% CI) *P-value*
**Mistreatment during birth**	**1.47(1.14–1.89)** ***0.003***	**1.48(1.14–1.92)** ***0.003***	**1.50(1.15–1.95)** ***0.002***	**1.50 (1.15–1.96)** ***0.003***
**Maternal age (in years)**				
≤19	**1.68(1.21–2.31)** ***0.002***	**1.72 (1.24–2.38)** ***0.001***		**1.72(1.23–2.41)** ***0.002***
20–29	Ref	Ref		Ref
≥30	1.03 (0.73–1.46) *0.873*	1.03(0.72–1.47) *0.868*		1.06 (0.74–1.52) *0.762*
**Maternal employment**				
No	Ref	Ref		Ref
Yes	1.10 (0.73–1.64) *0.644*	1.10(0.73–1.67) *0.647*		1.09(0.72–1.67) *0.680*
**Mode of birth**				
Vaginal	Ref			Ref
Emergency cesarean	0.84(0.60–1.17) *0.300*		0.80 (0.57–1.13) *0.207*	0.84(0.59–1.19) *0.320*
**Sex of infant**				
Male	Ref			Ref
Female	**1.31(1.01–1.68)** ***0.038***		**1.33 (1.03–1.72)** ***0.029***	**1.32(1.01–1.74)** ***0.039***
**Newborn complications**				
No	Ref			Ref
Yes	1.36(0.88–2.08) *0.165*		1.33 (0.85–2.08) *0.218*	1.39(0.88–2.16) *0.158*

^a^Block I= Socio-demographic factors (maternal age and maternal employment).

^b^Block II= current obstetric characteristics (mode of birth, sex of infant, newborn complications).

^c^Block III= Socio demographic factors + current obstetric characteristics.

^d^cRR= crude Relative Risk.

^e^aRR= adjusted Relative Risk.

## Discussion

The overall results of this study underline the increased likelihood of experiencing postpartum depressive symptoms if exposed to mistreatment around the time of childbirth in a health facility. Even after adjustment for socio-demographic and obstetric characteristics, the risk increased compared with those without exposure to mistreatment during childbirth.

In our study, the typology of mistreatment included verbal abuse, limited birth companionship, feeling ignored by health workers, feeling that their presence was a nuisance to health workers and their concerns or questions unanswered by health workers. Similar findings of inadequate information about care, less coverage of labour companionship, lack of opportunity to discuss concerns, and lack of positive discussion about pain relief were reported in the Nepal studies [46,47,50]. Despite the potential for normalization of mistreatment during childbirth in Nepal, there is a high prevalence of postpartum depression among Nepalese women who gave birth in health facilities [65–67]. The main finding here is that this study confirms the association of mistreatment during childbirth with postpartum depressive symptoms found in other studies [31–36]. Freedman’s bull’s eye framework suggests that in a given context, mistreatment during childbirth may be normalized by both providers and clients and accepted for better maternal and neonatal health outcomes [20,25,39]. National figures for Nepal indicate gender inequity [68], with only sixty-nine percent of women being educated and less economically active than men [55]. Violence against women is also widespread in Nepal [68,69]. There is evidence that gender inequality and high levels of tolerance of violence contribute to the social acceptance of mistreatment during childbirth in health facilities [25,45,70].

Within our cohort population, adolescent mothers and the women who gave birth to female infants exposed to mistreatment during childbirths were at relatively high risk of experiencing postpartum depressive symptoms. A recent community-based survey reported a similar finding that mistreatment during childbirth led to low service satisfaction among adolescent mothers [71]. Adolescent pregnancy in Nepal is associated with low socioeconomic status and obstetric complications [72,73]. A study in Nepal found high rates of depressive symptoms among adolescent mothers [74]. Exposure of such populations to mistreatment during childbirth could further exacerbate the vulnerability of adolescent mothers, leading to poor mental health outcomes. Similarly, the preference for male babies in Nepalese society has influenced reproductive behavior and the demographic structure of the nation [75,76]. This preference can lead women to blame themselves for giving birth to female babies and fear future pregnancies or sex-selective abortions, posing a threat to women’s mental health after childbirth [77].

Although Respectful Maternity Care (RMC) has been legally recognized in the Safe Motherhood and Reproductive Health Rights Act of Nepal (The Act), 2018, there is a transition gap from paper to action in providing safe and dignified maternity care [78]. Moreover, health care providers tend to justify their intentional or unintentional acts of mistreatment with patient overcrowding, work overload, structural deficiencies, and poor working conditions [20,79]. Lack of sleep, social isolation, constant hormonal changes, and the new responsibilities of motherhood can be overwhelming for women in the postpartum period [31]. The challenges of individual and social adjustment, coupled with the experience of mistreatment during childbirth, may exacerbate feelings of guilt and frustration in postnatal women, leading to the development of depressive symptoms [28]. It is important to identify and address the health facility determinants of common perinatal mental health problems, given their fatal consequences. The main finding of this study, confirming the association between mistreatment during childbirth and postpartum depressive symptoms, will add to the knowledge of postpartum mood disorders for appropriate early intervention at the health facility level.

The results of our study need to be interpreted with caution, given some methodological limitations. First, women who had a planned caesarean section were excluded from the study. We cannot ignore the possible contribution of caesarean section to the development of postpartum depressive symptoms. However, recent evidence suggests that mistreatment intensifies with the peak of labor [19], which requires interaction with providers during labor. Second, the data were collected by telephone interview, which allows for possible interference from family members, which may have led to under- or over-reporting. However, the telephone interview technique has been used for the EPDS in previous studies in Brazil and Nepal [35,66]. In addition, telephone interviews are a cost-effective method of data collection, which is particularly suitable when funding is limited [80]. Third, neutral responses to questions about mistreatment during childbirth are categorized as ‘agree’ to account for potential underreporting and social desirability bias due to fear of repercussions from health care providers and the normalization of mistreatment. We recognize that the neutral response may misinterpret true opinions. However, a similar analytical strategy was used in a cross-sectional study of direct observation and community-based surveys in four countries [19]. Fourth, although some studies suggest including all postpartum women, regardless of their mode of birth, we included only women who were in the active phase of labor pain during their hospital stay. This decision was based on the literature suggesting that mistreatment is common as labor progresses [19]. Fifth, although participants diagnosed with, or receiving treatment for, mental illness were excluded from the study, we cannot completely rule out the inclusion of participants with mental health problems that were not documented in the hospital records. We are also aware that women’s mental state at the time of the interview may have influenced their perception of mistreatment during childbirth, leading to misreporting. However, we believe that they had time to mentally process the birth events, as the assessment was conducted after the 6-week postpartum period had ended. Lastly, the study site is a referral hospital with its own complexities of management and service delivery, hence, the linkage between postpartum depressive symptoms and mistreatment during childbirth cannot be generalized.

Nevertheless, the strength of this study lies in the assessment of mistreatment during the postpartum period rather than immediately after birth, which indicates recall accuracy [31,81]. The literature suggests that a history of pre-existing psychiatric illness is an important contributor to postpartum depression [82]. However, women with mental illnesses were excluded from this study in an attempt to measure the risk of postpartum depressive symptoms in the general population of postpartum mothers. Therefore, the main findings of the study strengthen the possible association between mistreatment during childbirth and postpartum depressive symptoms. To our knowledge, this is the first study of its kind in Nepal to investigate the association between hospital birth mistreatment and postpartum depressive symptoms.

## Conclusion

Mistreatment during institutional childbirth has been identified as a potential contributor to depressive symptoms in postpartum women in a tertiary-level hospital in Nepal. Ensuring a respectful and caring birth environment for every woman should therefore be at the core of maternal and newborn health care provision. Urgent and responsive strategies are needed to implement tailored RMC interventions, particularly for adolescent mothers. Policy changes should be made to facilitate routine antenatal screening of birth expectations and fear of birth in pregnant women. Additionally, routine screening for common perinatal mental health problems is equally important for early identification and intervention. We recommend that additional observational studies be conducted to assess the immediate and long-term consequences of mistreatment during facility-based childbirth. Similarly, further exploratory studies are needed to understand the underlying power dynamics and complex interactions of mistreatment during childbirth.

## Abbreviations

EPDS: Edinburg Postnatal Depression Scale
cRR: crude Risk Ratio
aRR: adjusted Risk Ratio
MDD: Major Depressive Disorder
SD: standard deviations
CI: confidence intervals
RMC: Respectful Maternity Care

## Data Availability

The datasets generated and/or analyzed during the current study cannot be made publicly available due to the involvement of information of individuals disclosing identifiers.
